# Hematological and plasma profiles and ticks and tick-borne pathogens in wild Formosan black bears (*Ursus thibetanus formosanus*)

**DOI:** 10.1186/s13071-024-06320-7

**Published:** 2024-05-28

**Authors:** Yi-Lun Tsai, Wittawat Wechtaisong, Ting-Rong Lee, Chun-Hao Chang, Pin-Huan Yu, Mei-Hsiu Hwang

**Affiliations:** 1 Department of Veterinary Medicine, College of Veterinary Medicine, National Pingtung University of Science and Technology, Pingtung, Taiwan; 2 Veterinary Medical Teaching Hospital, College of Veterinary Medicine, National Pingtung University of Science and Technology, Pingtung, Taiwan; 3 Center of Excellence in Animal Vector-Borne Diseases, Veterinary Parasitology Unit, Department of Veterinary Pathology, Faculty of Veterinary Science, Chulalongkorn University, Bangkok, Thailand; 4 Institute of Wildlife Conservation, College of Veterinary Medicine, National Pingtung University of Science and Technology, Pingtung, Taiwan; 5 Institute of Veterinary Clinical Science, College of Veterinary Medicine, National Taiwan University, Taipei, Taiwan

**Keywords:** Hematological profile, Plasma biochemical profile, *Hepatozoon*, *Babesia*, Formosan black bear, Tick

## Abstract

**Background:**

The endangered Formosan black bear (*Ursus thibetanus formosanus*) is the largest native carnivorous mammal in Taiwan. Diseases, poor management, illegal hunting, and habitat destruction are serious threats to the survival of bear populations. However, studies on the impact of diseases on bear populations are limited. Therefore, this study aimed to establish a database of the hematological and plasma profiles of free-ranging Formosan black bears and investigate the occurrence of ectoparasites, blood parasites, and vector-borne pathogens.

**Methods:**

Formosan black bears were captured in Yushan National Park (YNP) and Daxueshan Forest Recreation Area (DSY) in Taiwan. Blood samples were collected from each bear for hematological analysis and plasma biochemistry using a hematology analyzer. Parasites and pathogens were detected using a thin blood smear with Wright–Giemsa staining and polymerase chain reaction (PCR) assay. Additionally, macroscopic ectoparasites were collected from bears to detect blood parasites and other pathogens. Moreover, the relationships between the bear variables (sex, age, and occurrence of parasites or pathogens), ectoparasites, and infectious agents were also analyzed.

**Results:**

In all, 21 wild bears (14 in YNP and 7 in DSY) were captured and released during the satellite tracking studies. Hematological analysis and plasma biochemistry indicated significant differences in white blood cells (WBC), segments, creatine kinase (CK), and lactate dehydrogenase (LDH) levels between foot snare and culvert-captured bears. Additionally, there were significant differences in total plasma protein (TPP), creatinine, Ca^2+^, Mg^2+^, and K^+^ levels between male and female bears. Moreover, pathogen-infected bears had significantly higher erythrocyte sedimentation rate (ESR; 30 min and 1 h) and globulin levels than uninfected bears. In total, 240 ticks were collected from 13 bears, among which eight adult tick species were identified, including *Haemaphysalis flava*, *Haemaphysalis hystricis*, *Amblyomma testudinarium*, *Ixodes ovatus*, *Dermacentor taiwanensis*, *Haemaphysalis longicornis*, *Ixodes acutitarsus*, *Amblyomma javanense*, and nymphs belonging to *Haemaphysalis* spp. PCR revealed that 13 (61.90%) and 8 (38.10%) bears harbored *Hepatozoon ursi* and *Babesia* DNA, respectively. Among the ticks examined, 157 (65.41%) and 128 (53.33%) samples were positive for *H. ursi* and *Babesia*, respectively.

**Conclusions:**

To the best of our knowledge, this is the first study to establish a database of the hematological and plasma profiles of wild Formosan black bears and investigate ectoparasite infestation and *Hepatozoon* and *Babesia* spp. infection. In conclusion, these findings may serve as a reference for monitoring the health and population of locally endangered bears.

**Graphical Abstract:**

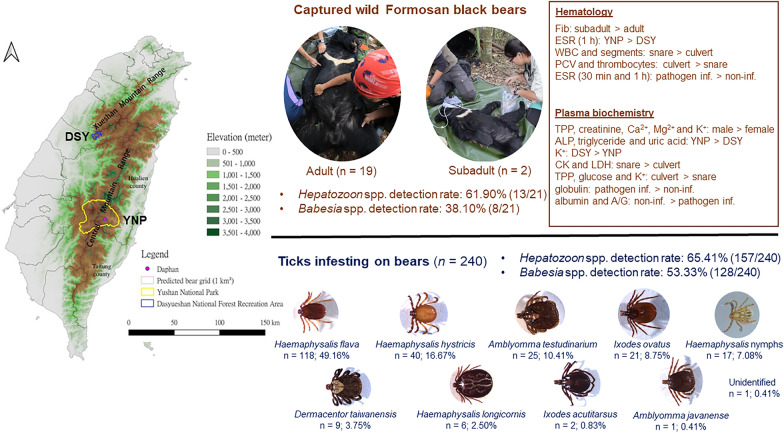

**Supplementary Information:**

The online version contains supplementary material available at 10.1186/s13071-024-06320-7.

## Background

Large carnivores perform vital ecological functions increasing their ecological integrity and biodiversity. The Formosan black bear (*Ursus thibetanus formosanus*) is a subspecies of Asiatic black bear and is indigenous to Taiwan. Presently, the Formosan black bear is classified as “Vulnerable” by the International Union for Conservation of Nature (IUCN) Red List of Threatened Species and listed in Additional file 1 of the Convention on International Trade in Endangered Species of Wild Fauna and Flora (CITES). Additionally, the Formosan black bear is the largest native carnivore in Taiwan, and is listed as an endangered species under Taiwan’s Wildlife Conservation Act. Notably, Formosan black bears function as umbrella and landscape species for conservation because of their wide home range [1].

Importantly, the conservation of bear populations needs to be considered in relation to several aspects, such as population structure, environment, genetics, disease, and management conditions. Habitat destruction and illegal hunting are critical threats to the survival of Formosan black bears [2]. Recent ecological studies on carnivores have shown that epidemic diseases seriously threaten several endangered wild animals, especially in populations with reduced numbers, malnutrition, and stress, or in inbreeding groups. However, little is known about the effects of diseases on the population of Formosan black bear. To prevent further decline in the already small bear population, research should focus on potentially threatening diseases and environmental conditions [3].

Blood and plasma biochemical reference values can assist clinical veterinarians in interpreting individual nutritional and physiological conditions and serve as the basis for disease diagnosis. For example, a comprehensive understanding of the physiology, hematology, and serum biochemistry of American black bears (*Ursus americanus*) and brown bears (*Ursus arctos*) is crucial for evaluating population health managing both captive and wild bear populations [4–8]. Additionally, serological studies have detected several pathogens in bears in America and Europe [9–12]. However, information on the hematology and serum biochemistry of Asiatic black bears is limited, except for a few studies on captive bears in Thailand, Taiwan, and South Korea [13–15].

A comprehensive understanding of pathogen exposures is important to assess the overall health of bear populations and reveal possible infectious agents that may affect the health of humans and other animals. Blood macro- and micro-parasites, such as heartworms, *Hepatozoon ursi*, and *Babesia* spp., have been detected in the blood samples of different bear species in several countries [16–20]. Zoonotic pathogens, including *Bartonella* spp., *Rickettsia* spp., and *Borrelia burgdorferi* s.l., have also been detected in bear populations [21, 22]. Considering that wild bears can act as reservoirs of various pathogens, some of which pose risks to human health via zoonotic transmission, identifying these pathogens may help prevent potential disease threats to human populations. Among ectoparasites, lice (*Trichodectes pinguis*) and ticks (*Haemaphysalis megaspinosa*) were found on a gunshot-wounded Asiatic black bear [23]. Additionally, *Demodex ursi* and various species of hard ticks (*Amblyomma americanum*, *Amblyomma maculatum*, *Dermacentor variabilis*, *Ixodes scapularis*, *Ixodes affinis*) were found on American black bears [24–26].

Although some physiological data are available [5, 6, 8, 27, 28], knowledge of potential infectious and vector-borne pathogens in Formosan black bears is still lacking. Therefore, this study aimed to establish a database of the hematological and plasma profiles of free-ranging Formosan black bears and investigate the occurrence of ectoparasites, blood parasites, and vector-borne pathogens. Overall, it is anticipated that this study will provide useful information for the conservation and management of endangered Formosan black bear populations.

## Methods

### Capture-release procedures for Formosan black bears

**Fig. 1:**
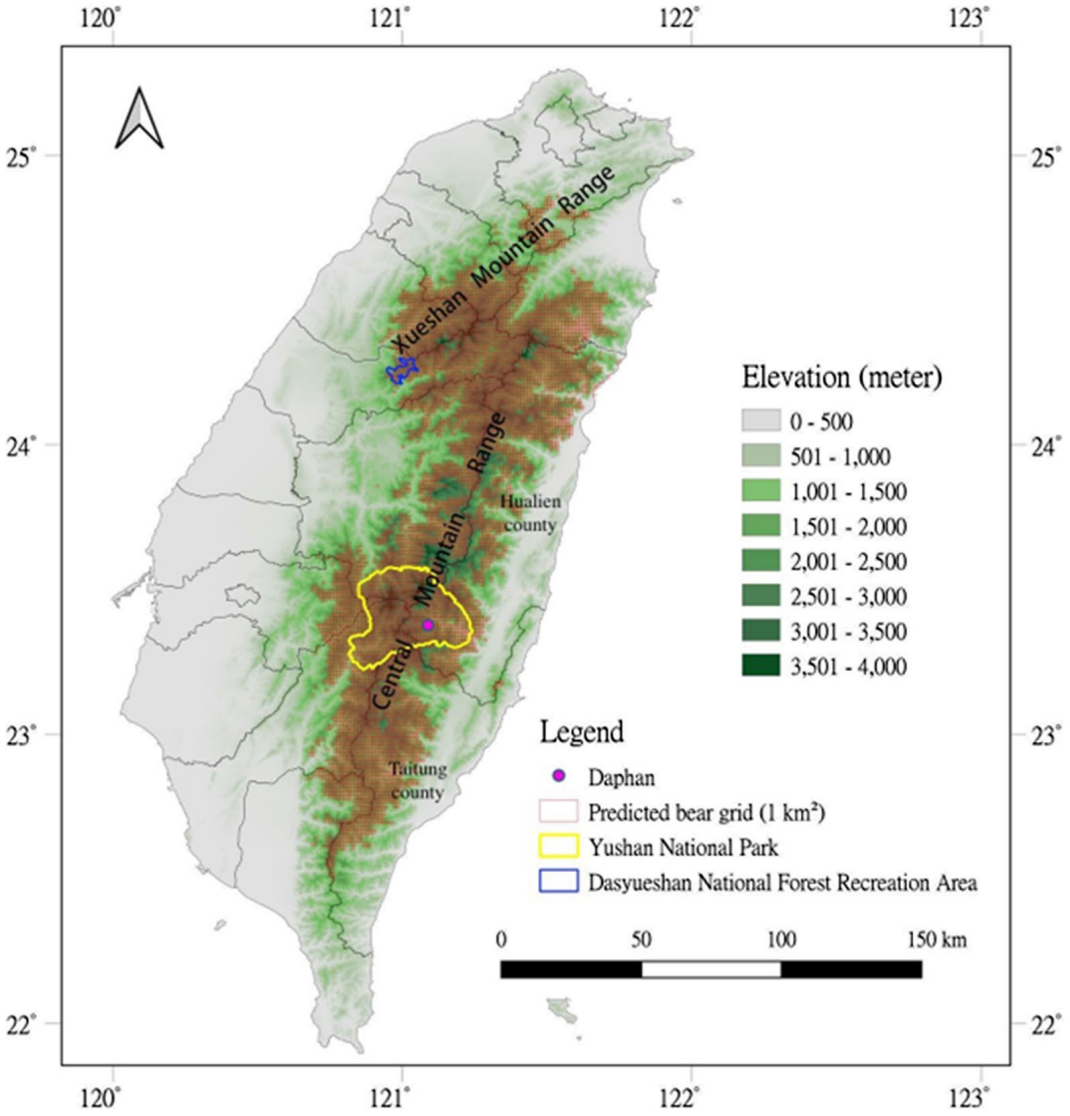
Two study sites of Formosan black bears (*Ursus thibetanus formosanus*) captured and released during 2014–2021—Yushan National Park (yellow line) and Daxueshan National Forest Recreation Area (blue line). The sites are distributed in the Central Mountain Range and Xueshan Mountain Range, respectively, of Taiwan. Red grids (1 × 1 km^2^) represent the predicted distribution of Formosan black bears [29]

Formosan black bears were captured from November 2014 to July 2021 at Yushan National Park (YNP) and Daxueshan Forest Recreation Area (DSY) in Taiwan for Global Positioning System (GPS) telemetry. YNP is located in the Central Mountain Range (23° 24′ 26.0604″ N, 121° 1′ 17.3604″ E), which covers an area of 1031.214 km^2^ (Fig. 1). DSY is located in the southeastern part of the Xueshan Mountain Range, near Taichung City (24° 15′ 11.16″ N, 121° 0′ 31.68″ E), covering an area of 39.63 km^2^ (Fig. 1). Notably, these areas have a relatively high bear density. All procedures involving bear capture, release, anesthetization, and sampling have been approved by the Forestry and Nature Conservation Agency of the Ministry of Agriculture under the Agreement of the Use of Conserved Wild Animals (No. 1031700938 and 1041701149) and the National Pingtung University of Science and Technology Laboratory Animal Care and Use Committee (IACUC No. NPUST-102-051 and NPUST-104-047).

**Fig. 2:**
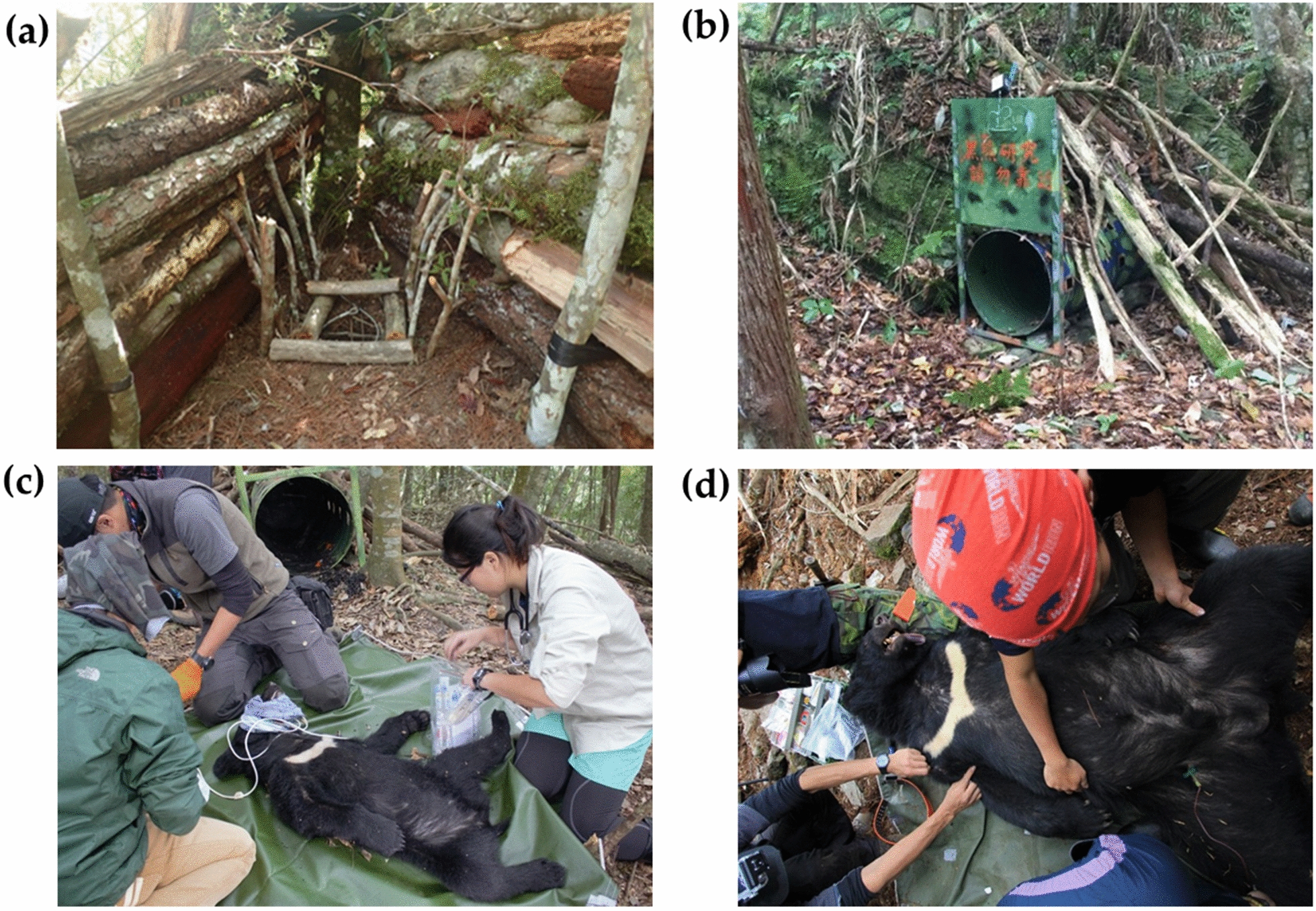
The setting of (**a**) snare and (**b**) culvert traps and sample collection for (**c**) subadult and (**d**) adult Formosan black bears

Black bears were captured using an Aldrich spring-activated foot snare [30] and culvert traps (L × W × H: 200 × 78 × 78 cm) [31] (Fig. 2a, b), with the former only applied in YNP where some sites had no road access and required a 3-day hike. Notably, both traps were used from November 2014 to May 2016, and only culverts were used afterwards. Captured bears were immobilized using mixed doses of different anesthetics based on the capture procedure and the individual bear status. For snare-captured and anxious bears, 0.03 mg/kg of dexmedetomidine (Dexdomitor®, Pfizer Inc., New York, NY) and 3.0 mg/kg of tiletamine-zolazepam (Zoletil®, Virbac Inc., Carros, France) were used. For small bears captured in Culvert traps, 0.025 mg/kg of dexmedetomidine and 2.5 mg/kg of tiletamine-zolazepam were used [32, 33]. Mixed anesthetics were delivered intramuscularly through blow darts.

After anesthetization, physical signs of anesthetic depth and physiological parameters, including body temperature and respiratory rate, were monitored and recorded. Pulse rate and blood oxygen saturation were monitored using a pulse oximeter (9847 V; NONIN Medical, Plymouth, MN, USA) with a sensor attached to the tongue. Individual sex, age, body weight, and morphometric characteristics were measured and recorded (Fig. 2c, d) [34]. Bear age was estimated and classified as adult or subadult on the basis of first premolar tooth sections or tooth wear [34, 35]. Bears aged 4 years or older were classified as adults [36]. After collection of blood samples and infesting ectoparasites, radio frequency identification (RFID) microchips were implanted into the hind necks of the bears, and ear tags were applied for individual identification. Additionally, GPS collars were attached to each bear for satellite tracking. At the end of the procedure, 10 mg of atipamezole (Antisedan®, Pfizer Inc., New York, NY) for each mg of dexmedetomidine were administered intramuscularly to reverse the anesthetic effect [37].

### Sample collection procedures

Blood samples (10–20 mL) were collected from the cephalic or femoral vein in ethylenediaminetetraacetic acid (EDTA) anticoagulant tubes (BD Biosciences, Franklin Lakes, NJ, USA) and stored at 4 °C in a portable refrigerator. Ectoparasites were collected from the skin using tweezers and stored in microcentrifuge tubes containing 70% ethanol [38]. All samples were transported within 3 days to the Veterinary Medical Teaching Hospital and the Laboratory of Public Health and Epidemiology, National Pingtung University of Science and Technology for morphological identification and laboratory processes.

### Thin blood smear, hematology, and plasma biochemistry examination

At least six thin Wright–Giemsa stained blood smears were obtained from each bear [39]. Microscopic examination was performed to explore the morphology of blood cells and identify the presence of blood parasites. Additionally, 14 hematological parameters were analyzed, including packed cell volume (PCV), total red blood cells (RBC), hemoglobin (Hb), mean corpuscular volume (MCV), mean corpuscular hemoglobin (MCH), mean corpuscular hemoglobin concentration (MCHC), total white blood cells (WBC), white blood cell types (segments, bands, lymphocytes, monocytes, eosinophils, basophils), and total platelet count (thrombocytes). White blood cell classifications were performed using an IDEXX ProCyte Dx Hematology analyzer instrument (IDEXX Laboratories Inc.,Westbrook, ME), whereas the remaining blood parameters were analyzed using the Mythic 18 Vet hematology analyzer instrument (Orphee SA, Switzerland). Additionally, erythrocyte sedimentation rate (ESR; 30 min and 1 h) and platelet fibrinogen (Fib) levels were analyzed using the Wintrobe method and heat precipitation fibrinogen test, respectively. The Fujifilm Dri-Chem 3500 s instrument (Fujifilm, Kanagawa, Japan) was used to measure the following 28 plasma biochemical parameters: total plasma protein (TPP), albumin, globulin, albumin/globulin ratio (A/G), bilirubin (T. Bil), aspartate aminotransferase (AST), alanine aminotransferase (ALT), alkaline phosphatase (ALP), gamma-glutamyl transferase (GGT), creatine kinase (CK), lactate dehydrogenase (LDH), cholesterol, triglyceride, glucose, amylase, lipase, creatinine, blood urea nitrogen (BUN), uric acid, free calcium (Ca^2+^), inorganic phosphorus (iP), and magnesium (Mg^2+^). Sodium (Na^+^), potassium (K^+^), and chloride (Cl^−^) electrolytes were analyzed using EasyLyte PLUS (Medica Corporation, Bedford, MA). Total carbon dioxide (TCO_2_), lactate, and plasma iron levels were determined using the Kodak Ektachem DT60 analytical system with DTE and DTSC II modules.

### Morphological identification of ectoparasites

The ectoparasites collected in this study were all ticks. Briefly, ticks were surface-rinsed twice with deionized water and observed under a dissecting microscope for morphological identification using taxonomic keys [40, 41]. After morphological identification, each tick was individually placed in a microcentrifuge tube containing 70% ethanol and stored at 4 °C for DNA extraction.

### DNA extraction from blood and tick samples of bears

Blood and tick samples were collected to detect parasitic and tick-borne pathogens. Tick samples were dissected in sterilized 1 × PBS. For nymphs, the whole body was cut and manually homogenized, whereas each adult tick was cut into approximately two halves. Half of each adult tick sample was transferred to a clean microcentrifuge tube for DNA extraction. A total of 100 µL of sterile 1 × PBS was added to each tube and stored at 4 °C until DNA extraction. The other half was stored in 70% ethanol as a backup. DNA was extracted from blood and tick samples using the DNeasy Blood and Tissue kit (Qiagen, Hilden, Germany), following the manufacturer’s instructions. All DNA samples were eluted into a 100 µL final volume and stored at −20 °C until used for blood parasite and pathogen detection.

### Polymerase chain reaction (PCR)

In this study, the presence of three selected blood parasites (*Dirofilaria ursi*, *Hepatozoon* spp., and *Babesia* spp.) [17, 42, 43] and three zoonotic pathogens (*Bartonella* spp., *Rickettsia* spp., and *Borrelia burgdorferi* s.l.) [21, 22] in blood and tick samples was determined using PCR. PCR was performed using 20 µL of reaction mixture containing 5 µL of DNA template, 0.5 µL of 10 µM of each primer, 10 µL of 2 × Taq buffer (Bio-GENESIS Q-AMPTM 2 × Screening Fire Tag Mix kit, Illumina Co., USA), and distilled water. The primers and PCR conditions used for blood parasite and pathogen detection are listed in Table 1. DNA from each blood parasite, pathogen (positive control), and distilled water (negative control) were used for the PCR assay. Finally, PCR products were separated using a 2% agarose gel stained with nucleic acid stain (50 ppm EtB “out” nucleic acid staining solution, Yeastern Biotech., Taiwan), and the results were visualized with UVIdoc HD5 (Uvitec, Cambridge, UK).

**Table 1:** Primers for detecting blood parasites and pathogens of wild Formosan black bears used in this study

Blood parasites/pathogens	Primers	5′–3′	Target size (bp.)	References
*Dirofilaria ursi*	WSPintF	TTAGACTGCTAAAGTGGAATT	378	[44]
	WSPestR	AAACCACTGGGATAACAAGA		
*Hepatozoon* spp.	HepF	ATACATGAGCAAAATCTCAAC	667	[45]
	HepR	CTTATTATTCCATGCTGCAG		
*Babesia* spp.	BabF	GTTTCTGMCCCATCAGCTTGAC	422–440	[46]
	BabR	CAAGACAAAAGTCTGCTTGAAAC		
*Bartonella* spp.	BhCS.781p	GGGGACCAGCTCATGGTGG	380–400	[47]
	BhCS.1137n	AATCGAAAAAGAACAGTAAACA		
*Rickettsia* spp.	Rp.877p	GGGGACCTGCTCACGGCGG	381	[48]
	Rp.1258n	ATTGCAAAAAGTACAGTGAACA		
*Borrelia burgdorferi* s.l.	flaB-Outer 1	AARGAATTGGCAGTTCAATC	497	[22]
	flab-Outer 2	GCATTTTCWATTTTAGCAAGTGATG		

### Sequencing and statistical analyses

PCR products were purified using a Plus DNA Clean/Extraction Kit (GMbiolab Co., Ltd., Taichung, Taiwan) and sent for nucleotide sequencing (Genomics BioScience and Technology Co., Ltd., Taiwan). The sequence data were compared with known sequences deposited in the GenBank database using the National Center for Biotechnology Information (NCBI) nucleotide Basic Local Alignment Search Tool (BLAST). For genetic analysis, the validated sequences were aligned and analyzed using MegAlign (DNASTAR, Inc., Madison, WI, USA).

All statistical analyses were performed using GraphPad Prism version 8.4.2. The numerical data of each blood parameter were summarized as medians, means, and standard deviations. Comparisons of blood parameters between each bear variable of the bears, such as sex, age, area, trap type, and pathogen occurrence, were performed using unpaired *t*-tests for parametric data and Mann–Whitney *U* tests for non-parametric data. Additionally, multiple comparisons of blood parameters among pathogen infection, co-infection, and non-infection statuses of the bears were performed using the Kruskal–Wallis test, and *P* < 0.05 was considered statistically significant.

The relationships between bear variables (sex, age, and area) and blood parasite and pathogen infection status; tick variables (sex, life stage, and area) and pathogen harboring status; and bear variables (sex, age, and occurrence of blood parasites and pathogens) and the presence of blood-parasite- and pathogen-carrying ticks were analyzed using the chi-square test. A two-tailed Fisher’s exact test was used when the expected number of observations was less than five, and *P* < 0.05 was considered statistically significant.

## Results

### Demography of captured Formosan black bears

In total, 21 Formosan black bears were successfully captured and released (14 in YNP and 7 in DSY) between November 2014 and July 2021. Among the bears captured, there were 13 adults (seven males and six females) and one male subadult in YNP and six adults (three males and three females) and one male subadult in DSY. Blood samples were successfully collected from all bears, and ectoparasites were collected from 13 bears (61.90%) (Additional file 1: Table S1). Additionally, five bears were captured by snares in 2014–2015, while the rest were captured by culvert traps in 2015–2021.

### Hematological and plasma profiles of captured Formosan black bears

The hematological and plasma biochemical profiles of wild Formosan black bears are summarized in Tables 2 and 3. TPP, creatinine, Ca^2+^, Mg^2+^, and K^+^ levels were higher in male bears than in females, and Fib value was higher in subadults than in adults. Additionally, ESR (1 h), ALP, triglycerides, and uric acid levels were higher in bears captured in YNP than in those captured in DSY, whereas the K^+^ value was higher in bears captured in DSY. Moreover, WBC, segment, CK, and LDH values were higher in snare-captured bears than in culvert-captured bears, whereas PCV, thrombocytes, TPP, glucose and K^+^ values were higher in culvert-captured bears. Regarding pathogen-infection-related differences, both ESR (30 min and 1 h) and globulin values were higher in infected bears than in non-infected bears, whereas albumin and A/G values were higher in non-infected bears. Furthermore, we compared blood parameters among bears infected with *Babesia* spp. (*n* = 2) and/or *H. ursi* (*n* = 7), co-infected with *Babesia* spp. and *H. ursi* (*n* = 6) and uninfected with the parasites (*n* = 6). Notably, there were significant differences in ESR (1 h; *P* = 0.0163), albumin (*P* = 0.0100), globulin (*P* = 0.0454), A/G (*P* = 0.0058), ALP (*P* = 0.0313), and Ca^2+^ (*P* = 0.0121) values among groups (Tables 4, 5). Multiple comparisons showed that albumin and A/G values were higher in non-infected bears than in bears co-infected with *Babesia* spp. and *H. ursi* (*P* = 0.0077 and *P* = 0.0233, respectively). Additionally, *Babesia*-infected bears had significantly higher (*P* = 0.0453) globulin levels and lower (*P* = 0.0278) A/G value than uninfected bears. Moreover, ALP and Ca^2+^ levels were higher in *H. ursi*-infected bears than in uninfected (*P* = 0.0327) and co-infected (*P* = 0.0314) bears (Table 5).

**Table 2:** Hematological profiles and the variation by bear sex, age, captured area, trap type, and occurrence of pathogens in wild Formosan black bears

Parameter	Mean	Median	SD	Sex			Age			Area			Trap			Pathogen detection by PCR		
				Male (*n* = 12)	Female (*n* = 9)	*P*-value	Adult (*n* = 19)	Subadult (*n* = 2)	*P*-value^*^	YNP (*n* = 14)	DSY (*n* = 7)	*P*-value^*^	Snare (*n* = 5)	Culvert (*n* = 16)	*P*-value^*^	Positive (*n* = 15)	Negative (*n* = 6)	*P*-value^*^
PCV (%)	39.21	38.10	6.36	41.35	36.37	0.0745	39.11	40.20	0.6952	38.40	40.82	0.4248	33.62	40.96	**0.0199**	38.62	40.70	0.5124
RBC (10^6^/μL)	5.75	5.56	1.06	6.01	5.40	0.2070	5.75	5.78	0.9524	5.64	5.93	0.5935	4.99	5.99	0.0651	5.72	5.82	0.8580
Hb (g/dL)	12.94	12.50	2.17	13.53	12.17	0.1586	12.91	13.25	0.9476	12.45	13.94	0.1414	11.32	13.46	0.0521	12.72	13.51	0.4618
MCV (fL)	68.14	69.00	4.83	68.84	67.22	0.4221	67.94	70.05	0.6857	67.80	68.82	0.5353	67.10	68.47	0.3539	67.51	69.73	0.5693
MCH (pg)	23.15	22.90	2.75	22.70	23.74	0.9861	23.16	23.00	0.8286	22.95	23.54	0.1655	22.96	23.21	0.7953	23.10	23.28	0.3913
MCHC (g/dL)	33.35	33.20	2.01	33.03	33.79	0.4046	33.40	32.90	0.6810	32.85	34.35	0.1082	34.26	33.07	0.2596	33.33	33.40	0.9476
WBC (μL)	11,153.33	9900.00	5114.76	10,735.83	11,710.00	0.8479	11,295.78	9800.00	0.9429	12,179.28	9101.42	0.2011	16,530.00	9473.12	**0.0107**	12,114.67	8750	0.2281
Segments (μL)	8068.95	7128.0	4739.35	7568.25	8736.55	0.5893	8151.78	7282.00	0.9524	9233.92	5739.00	0.1490	13,926.00	6238.62	**0.0003**	9212.26	5210.67	0.0798
Bands (μL)	49.04	0	137.04	57.58	37.67	0.5203	48.15	57.50	0.2714	24.21	98.71	0.0673	67.80	43.18	0.8830	58.93	24.33	0.5439
Lymphocytes (μL)	2494.38	1404.00	2967.75	2583.16	2376.00	0.3100	2602.21	1470.00	0.9524	2335.28	2812.57	0.7433	1781.00	2717.31	0.9681	2300.73	2978.50	0.7333
Monocytes (μL)	492.33	425.00	390.88	466.83	526.33	1.0000	454.47	852.00	0.4667	491.00	495.00	0.8557	733.00	417.12	0.2398	497.33	479.83	0.6222
Eosinophils (μL)	73.76	42.00	99.70	105.75	31.11	0.1125	66.94	138.50	0.0762	93.57	34.14	0.2666	18.20	91.12	0.0933	80.40	57.16	0.8423
Basophils (μL)	0	0	0	0	0	1.0000	0	0	1.0000	0	0	1.0000	0	0	1.0000	0	0	1.0000
Thrombocytes (10^3^/μL)	332.76	336.00	134.27	358.16	298.90	0.3293	342.52	240.00	0.4000	299.42	399.42	0.1092	168.40	384.12	**0.0012**	303.20	406.67	0.2051
ESR (30 min)	1.07	1.00	1.04	0.81	1.43	0.2828	1.14	0.50	0.5906	1.34	0.50	0.1017	1.60	0.89	0.0544	1.38	0.41	**0.0482**
ESR (1 h)	8.53	2.50	16.59	8.72	8.27	0.3381	9.10	3.75	0.7544	11.93	1.16	**0.0152**	6.94	9.10	0.2289	11.97	1.08	**0.0074**
Fib (g/dL)	0.33	0.20	0.23	0.34	0.32	0.5172	0.30	0.65	**0.0286**	0.31	0.37	0.9950	0.24	0.36	0.3500	0.32	0.36	0.9224

SD, standard deviation

^*^Significant values (*P* < 0.05) are marked with bold font

**Table 3:** Plasma biochemical profiles and the variation by bear sex, age, captured area, trap type, and occurrence of pathogens in wild Formosan black bears

Parameter	Mean	Median	SD	Sex			Age			Area			Trap			Pathogen detection by PCR		
				Male (*n* = 12)	Female (*n* = 9)	*P*-value^*^	Adult (*n* = 19)	Subadult (*n* = 2)	*P*-value	YNP (*n* = 14)	DSY (*n* = 7)	*P*-value^*^	Snare (*n* = 5)	Culvert (*n* = 16)	*P*-value^*^	Positive (*n* = 15)	Negative (*n* = 6)	*P*-value^*^
TPP (g/dL)	7.50	7.60	0.73	7.81	7.08	**0.0197**	7.57	6.85	0.1810	7.55	7.41	0.6994	6.80	7.72	**0.0095**	7.48	7.55	0.8631
Albumin (g/dL)	3.35	3.30	0.59	3.47	3.20	0.3062	3.36	3.25	0.9333	3.21	3.64	0.1220	2.92	3.49	0.0573	3.17	3.81	**0.0207**
Globulin (g/dL)	4.26	4.20	0.67	4.34	4.15	0.5180	4.33	3.60	0.1857	4.33	4.11	0.4907	3.88	4.38	0.1455	4.47	3.73	**0.0182**
A/G	0.81	0.83	0.23	0.84	0.78	0.5790	0.80	0.90	0.5190	0.76	0.91	0.1586	0.76	0.83	0.5544	0.72	1.04	**0.0018**
T. Bil (mg/dL)	0.46	0.40	0.28	0.41	0.52	0.4157	0.48	0.20	0.1619	0.49	0.40	0.4963	0.70	0.38	0.0743	0.49	0.38	0.4789
AST (U/L)	125.14	76.00	137.30	152.00	89.33	0.4116	115.57	216.00	0.9476	129.77	115.88	0.7014	223.16	94.51	0.1485	127.78	118.53	0.6340
ALT (U/L)	27.57	22.00	11.73	31.25	22.66	0.1065	27.26	30.50	0.4905	26.78	29.14	0.4317	32.40	26.06	0.4591	27.13	28.67	0.4350
ALP (U/L)	69.85	63.00	35.02	71.00	68.33	0.8681	66.21	104.50	0.7714	82.57	44.42	**0.0143**	75.40	68.12	0.3539	78.26	48.83	0.0548
GGT (U/L)	39.23	26.00	48.47	54.00	19.56	0.0709	41.78	15.00	0.1524	42.92	31.85	0.8409	19.20	45.50	0.1478	40.13	37.00	0.6093
CK (U/L)	4224.47	330.00	10,273.00	3957.41	4580.56	0.9170	3971.26	6630.00	0.9524	5252.57	2168.28	0.4430	14,210.40	1103.87	**0.0012**	4950.20	2410.16	0.6768
LDH (U/L)	999.42	692.00	820.71	985.16	1018.44	0.8769	1022.73	778.00	0.8667	1113.07	1022.73	0.2028	1829.60	740.00	**0.0415**	1117.60	704.00	0.3900
Cholesterol (mg/dL)	320.85	308.00	63.46	342.67	291.78	0.0674	327.57	257.00	0.1857	317.35	327.85	0.7306	308.80	324.62	0.6387	312.40	342.00	0.2411
Triglyceride (mg/dL)	348.28	314.00	120.49	376.08	311.22	0.3824	356.05	274.50	0.2381	381.50	281.85	**0.0097**	305.20	361.75	0.4451	369.13	296.16	0.1781
Glucose (mg/dL)	156.71	148.00	41.61	154.67	159.44	0.8021	160.31	122.50	0.3905	154.78	160.57	0.7724	124.40	166.81	**0.0433**	149.93	173.67	0.2475
Amylase (U/L)	50.56	17.00	85.41	24.65	85.11	0.2253	50.47	51.45	0.9333	42.74	66.21	0.7573	76.28	42.53	0.1720	39.06	79.31	0.6911
Lipase (U/L)	35.85	33.00	25.30	34.20	38.06	0.6131	37.82	17.20	0.1810	41.80	23.97	0.1429	30.64	37.48	0.5607	39.37	27.06	0.3295
Creatinine (mg/dL)	1.01	1.00	0.39	1.16	0.82	**0.0461**	0.99	1.25	0.6190	1.08	0.88	0.2904	0.88	1.06	0.3854	1.02	1.00	0.9314
BUN (mg/dL)	12.47	5.70	20.17	13.67	10.87	0.2392	9.37	41.90	0.8762	8.84	19.72	0.0639	15.80	11.43	0.9222	9.24	20.53	0.0766
Uric acid (mg/dL)	4.70	1.00	12.61	2.82	7.20	0.7138	3.98	11.50	0.3381	5.19	3.71	**0.0203**	12.34	2.31	0.4050	4.85	4.31	0.3481
Ca^2+^ (mg/dL)	6.83	7.30	2.11	7.54	5.88	**0.0389**	6.70	8.10	0.4048	6.58	7.32	0.4620	6.18	7.03	0.2000	6.35	8.03	0.1009
iP (mg/dL)	4.64	4.50	1.59	4.91	4.28	0.3866	4.58	5.25	0.8476	4.49	4.95	0.5440	4.80	4.60	0.8141	4.68	4.55	0.8647
Mg^2+^ (mg/dL)	1.64	1.60	0.26	1.74	1.51	**0.0493**	1.66	1.45	0.2048	1.65	1.61	0.9539	1.50	1.68	0.1556	1.64	1.65	0.5740
Na^+^ (mEq/L)	141.92	142.00	6.73	142.90	140.63	0.4597	141.81	143.00	0.8714	141.25	143.28	0.5279	140.30	142.43	0.5495	142.03	141.67	0.9137
K^+^ (mEq/L)	4.11	4.20	0.58	4.35	3.81	**0.0337**	4.05	4.70	0.2762	3.93	4.48	**0.0398**	3.33	4.36	**0.0001**	3.99	4.43	0.1239
Cl^−^ (mEq/L)	105.59	112.00	12.58	108.20	102.10	0.2118	105.91	102.50	0.8333	107.17	102.42	0.4317	107.68	104.93	0.8257	104.76	107.67	0.9549
TCO_2_ (mmol/L)	27.62	28.50	5.90	26.67	28.20	0.7509	27.28	30.00	N/A	26.83	30.00	0.5531	26.00	30.33	0.2143	27.28	30.00	N/A
Lactate (mg/dL)	5.39	4.40	4.32	4.44	6.71	0.8401	5.50	4.50	0.7485	5.32	5.55	0.2808	3.74	5.98	0.1290	5.29	5.61	0.1509
Plasma iron (μg/dL)	356.90	315.00	175.97	340.00	366.57	0.8238	319.60	730.00	N/A	332.87	421.00	0.4888	352.40	360.67	0.7922	319.60	730.00	N/A

N/A, Not applicable; SD, standard deviation

^*^Significant values (*p* < 0.05) are marked with bold font

**Table 4:** Comparison of hematological profiles and pathogen occurrence variation in wild Formosan black bears

Parameter	Mean	Median	SD	Occurrence of pathogen				
				*H. ursi* (*n* = 7)	*Babesia* spp. (*n* = 2)	Co-infection (*n* = 6)	Non-infection (*n* = 6)	*P*-value^*^
PCV (%)	39.21	38.10	6.36	41.70	41.55	34.05	40.70	0.1347
RBC (10^6^/μL)	5.75	5.56	1.06	6.24	6.36	4.91	5.82	0.0648
Hb (g/dL)	12.94	12.50	2.17	13.37	14.50	11.36	13.51	0.1720
MCV (fL)	68.14	69.00	4.83	66.77	65.65	69.00	69.73	0.8475
MCH (pg)	23.15	22.90	2.75	21.51	22.80	25.05	23.28	0.0736
MCHC (g/dL)	33.35	33.20	2.01	32.32	35.10	33.91	33.40	0.3422
WBC (μL)	11,153.33	9900.00	5114.76	10,100.00	9155.00	15,451.67	8750.00	0.1421
Segments (μL)	8068.95	7128.0	4739.35	7015.00	6851.00	12,562.83	5210.67	0.1269
Bands (μL)	49.04	0	137.04	0	272.50	56.50	24.33	0.2868
Lymphocytes (μL)	2494.38	1404.00	2967.75	2600.42	1902.50	2083.83	2978.50	0.8488
Monocytes (μL)	492.33	425.00	390.88	366.00	364.00	695.00	479.83	0.5788
Eosinophils (μL)	73.76	42.00	99.70	118.71	37.00	50.16	57.16	0.4943
Basophils (μL)	0	0	0	0	0	0	0	N/A
Thrombocytes (10^3^/μL)	332.76	336.00	134.27	341.14	415.50	221.50	406.67	0.0658
ESR (30 min)	1.07	1.00	1.04	1.07	0.50	2.00	0.41	0.0582
ESR (1 h)	8.53	2.50	16.59	14.00	1.00	11.34	1.08	**0.0163**
Fib (g/dL)	0.33	0.20	0.23	0.37	0.20	0.30	0.36	0.7392

N/A, not applicable; SD, standard deviation

^*^Significant values among groups (*P* < 0.05) are marked with bold font

**Table 5:** Comparison of plasma biochemical profiles and pathogen occurrence variation in wild Formosan black bears

Parameter	Mean	Median	SD	Occurrence of pathogen				
				*H. ursi* (*n* = 7)	*Babesia* spp. (*n* = 2)	Co-infection (*n* = 6)	Non-infection (*n* = 6)	*P*-value^*^
TPP (g/dL)	7.50	7.60	0.73	7.81	7.20	7.20	7.55	0.5390
Albumin (g/dL)	3.35	3.30	0.59	3.51^a,b^	3.10^a,b^	2.80^a^	3.81^b^	**0.0100**
Globulin (g/dL)	4.26	4.20	0.67	4.30^a,b^	5.30^a,b^	4.40^a^	3.73^b^	**0.0454**
A/G	0.81	0.83	0.23	0.84^a,b,c,d^	0.53^a,b,c^	0.65^a,b,c^	1.04^d^	**0.0058**
T. Bil (mg/dL)	0.46	0.40	0.28	0.52	0.35	0.50	0.38	0.7904
AST (U/L)	125.14	76.00	137.30	83.14	84.00	194.46	118.53	0.7134
ALT (U/L)	27.57	22.00	11.73	25.28	26.50	29.50	28.67	0.8508
ALP (U/L)	69.85	63.00	35.02	100.14^a^	52.00^ab^	61.50^ab^	48.83^b^	**0.0313**
GGT (U/L)	39.23	26.00	48.47	65.28	13.50	19.67	37.00	0.1162
CK (U/L)	4224.47	330.00	10,273.00	321.71	523.50	11,825.67	2410.16	0.2710
LDH (U/L)	999.42	692.00	820.71	726.42	1023.50	1605.33	704.00	0.5516
Cholesterol (mg/dL)	320.85	308.00	63.46	330.42	275.00	303.83	342.00	0.2953
Triglyceride (mg/dL)	348.28	314.00	120.49	423.71	277.50	336.00	296.16	0.1863
Glucose (mg/dL)	156.71	148.00	41.61	154.71	150.00	144.33	173.67	0.7574
Amylase (U/L)	50.56	17.00	85.41	20.62	12.30	69.50	79.31	0.0950
Lipase (U/L)	35.85	33.00	25.30	48.74	19.20	35.16	27.06	0.5415
Creatinine (mg/dL)	1.01	1.00	0.39	1.19	0.75	0.91	1.00	0.3650
BUN (mg/dL)	12.47	5.70	20.17	12.54	9.10	5.45	20.53	0.3571
Uric acid (mg/dL)	4.70	1.00	12.61	9.08	0.80	1.26	4.31	0.1431
Ca^2+^ (mg/dL)	6.83	7.30	2.11	7.97^a^	5.80^a,b^	4.65^b^	8.03^a,b^	**0.0121**
iP (mg/dL)	4.64	4.50	1.59	4.90	6.35	3.88	4.55	0.2587
Mg^2+^ (mg/dL)	1.64	1.60	0.26	1.81	1.50	1.48	1.65	0.1484
Na^+^ (mEq/L)	141.92	142.00	6.73	141.71	142.50	142.25	141.67	0.9890
K^+^ (mEq/L)	4.11	4.20	0.58	4.20	4.40	3.61	4.43	0.1455
Cl^−^ (mEq/L)	105.59	112.00	12.58	105.42	88.00	109.56	107.67	0.3397
TCO_2_ (mmol/L)	27.62	28.50	5.90	27.00	30.00	26.80	30.00	1.0000
Lactate (mg/dL)	5.39	4.40	4.32	6.85	4.20	3.32	5.61	0.1525
Plasma iron (μg/dL)	356.90	315.00	175.97	523.50	266.50	269.33	730.00	0.0605

SD, standard deviation

^*^Significant values (*P* < 0.05) among groups are marked with bold font

^a–d^Among rows: the values with different superscript letters in a row are significantly different (*P* < 0.05)

### Diversity of ticks collected from Formosan black bears

In total, 240 ectoparasites (all ticks) were collected from 13 bears. Notably, eight species of adult ticks were identified, including 118 (49.16%) *Haemaphysalis flava*, 40 (16.67%) *Haemaphysalis hystricis*, 25 (10.41%) *Amblyomma testudinarium*, 21 (8.75%) *Ixodes ovatus*, 9 (3.75%) *Dermacentor taiwanensis*, 6 (2.5%) *Haemaphysalis longicornis*, 2 (0.83%) *Ixodes acutitarsus*, 1 (0.41%) *Amblyomma javanense*, and 17 (7.08%) nymphs belonging to *Haemaphysalis* spp. (Table 6). Additionally, one tick was not identified owing to damage to its mouth and body during collection (Table 6).

**Table 6:** Numbers of ticks in different species collected from Formosan black bears and the proportion of ticks that tested PCR-positive for blood parasites

Tick species	Number of ticks (%)	Number of ticks positive for *H. ursi* (%)	Number of ticks positive for *Babesia* spp. (%)
*H. flava*	118 (49.16) [M: 49; F: 69]	89 (75.42) [M: 34; F: 55]	69 (58.47) [M: 28; F: 41]
*H. hystricis*	40 (16.67) [M: 13; F: 27]	18 (4.50) [M: 2; F: 16]	17 (4.25) [M: 6; F: 11]
*A. testudinarium*	25 (10.41) [M: 17; F: 8]	7 (28.0) [M: 4; F: 3]	7 (28.0) [M: 5; F: 2]
*I. ovatus*	21 (8.75) [M: 5; F: 16]	15 (71.42) [M: 2; F: 13]	10 (47.61) [M: 2; F: 8]
*D. taiwanensis*	9 (3.75) [M: 3; F: 6]	7 (77.77) [M: 2; F: 5]	9 (100.0) [M: 3; F: 6]
*H. longicornis*	6 (2.5) [M: 4; F: 2]	3 (50.0) [M: 1; F: 2]	0 (0)
*I. acutitarsus*	2 (0.83) [M: 0; F: 2]	2 (100.0) [M: 0; F: 2]	1 (50.0) [M: 0; F: 1]
*A. javanense*	1 (0.41) [M: 1; F: 0]	1 (100.0) [M: 1; F: 0]	1 (100.0) [M: 1; F: 0]
*Haemaphysalis* spp.	17 (7.08) [N: 17]	14 (82.35) [N: 14]	13 (76.47) [N: 13]
Unidentified	1 (0.41) [U: 1]	1 (100.0) [U: 1]	1 (100.0) [U: 1]
Total	240 (100.0) [M: 92; F: 130; N: 17; U: 1]	157 (65.41) [M: 46; F: 96; N: 14; U: 1]	128 (53.33) [M: 45; F: 69; N: 13; U: 1]

M, male; F, female; N, nymph; U, unidentified

### Blood parasite and pathogen detection in bears and ticks

**Fig. 3:**
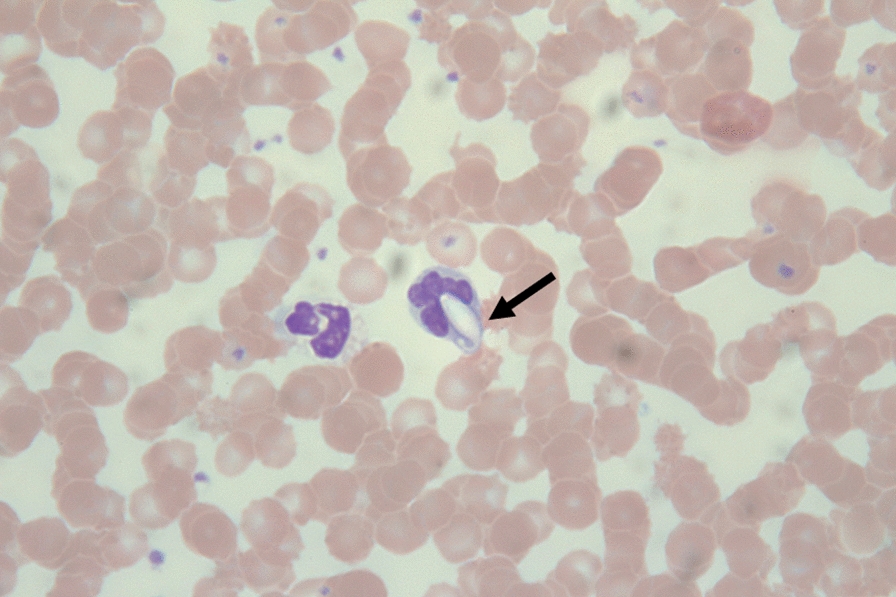
The beak-like protrusion at one end of the slightly curved gamont of *H. ursi* (arrowed) found in thin blood smears of Formosan black bears

*Hepatozoon* spp. gamonts were observed microscopically in thin blood smears (Fig. 3). Based on the morphology, the beak-like protrusion at one end of the slightly curved gamont is one of the most characteristic morphological features of *H. ursi*. PCR showed that 13 (61.90%) bears harbored *Hepatozoon* DNA. Additionally, DNA fragments from 10 random *Hepatozoon*-positive samples showed 99.1–99.7% similarity with the *H. ursi* isolate (EU041718) listed in the GenBank database. Although no *Babesia* spp. were observed in thin blood smears, *Babesia* DNA fragments were detected in eight (38.10%) blood samples and showed 90.6–94.4% similarity with *Babesia gibsoni* (KF171473), *Babesia microti* (JX962779), *Babesia odocoilei* (KC460321), and *Babesia* spp. (KC465978). Additionally, *H. ursi* and *Babesia* spp. co-infection was observed in six (28.57%) bears (all captured in YNP). However, PCR showed that all blood samples tested negative for *D. ursi*, *Bartonella* spp., *Rickettsia* spp., and *B. burgdorferi* s.l. DNA.

Among the 240 collected ticks, PCR showed that 157 (65.41%) were positive for *Hepatozoon* DNA (Table 6). DNA sequencing indicated that 50 randomly selected *Hepatozoon*-PCR-positive samples showed 99.0% similarity with the *H. ursi* isolate (EU041718) listed in the GenBank database. Importantly, 128 (53.33%) ticks were *Babesia*-positive, 40 of which were randomly selected for sequencing (Table 4). Additionally, the sequences showed 93.4–95.5% similarity with *B. gibsoni* (KF171473), *B. microti* (JX962779), and *B. odocoilei* (KC460321) isolates listed in the GenBank database. The results of PCR for *Hepatozoon* spp. and *Babesia* spp. in each tick species is shown in Table 4. Moreover, all tick samples tested negative for *D. ursi*, *Bartonella* spp., *Rickettsia* spp., and *B. burgdorferi* s.l. DNA using PCR.

### *H. ursi* and *Babesia* spp. infection in Formosan black bears and their ticks

Notably, the prevalence rates of *H. ursi* and *Babesia* spp. infection in Formosan black bears were 61.90% (13/21) and 38.10% (8/21), respectively. Additionally, the prevalence of *H. ursi* infection in bears captured in YNP (92.85%) was significantly higher (*P* = 0.0001) than that in bears captured in DSY (0%); however, there was no significant difference in sex or age (Table 5). Moreover, there were no significant differences in the prevalence of *Babesia* spp. infection among bears of different sexes, ages, and areas (Table 7).

**Table 7:** Numbers of *H. ursi*- and *Babesia* spp.-PCR-positive samples in different variables of wild Formosan black bears and ticks

	*H. ursi*		*Babesia* spp.	
	Number of positive samples and samples tested (%)	*P*-value^*^	Number of positive samples and samples tested (%)	*P*-value^*^
Variables of bears				
Sex				
Male	7/12 (58.33)	0.6972	3/12 (25.00)	0.1536
Female	6/9 (66.67)		5/9 (55.56)	
Age				
Adult	12/19 (63.15)	1.0000	8/19 (42.10)	0.5048
Subadult	1/2 (50.00)		0/2 (0)	
Area				
YNP	13/14 (92.85)	0.0001	6/14 (42.85)	0.5251
DSY	0/7 (0)		2/7 (28.57)	
Variables of ticks				
Sex				
Male	46/92 (50.0)	**0.0002**	45/92 (48.91)	0.5408
Female	96/130 (73.84)		69/130 (53.07)	
Life stage				
Adult	142/222 (63.96)	0.1856	114/222 (51.35)	0.0749
Nymph	14/17 (82.35)		13/17 (76.47)	
Area				
YNP	139/215 (64.65)	0.4646	109/215 (50.70)	**0.0014**
DSY	18/25 (72.0)		21/25 (84.00)	

^*^Significant values (*P* < 0.05) are marked with bold font

Furthermore, the prevalence rates of *H. ursi* and *Babesia* spp. infections in ticks collected from the bears were 65.41% (157/240) and 53.33% (128/240), respectively. Adult female ticks (73.84%) had a significantly higher (*P* = 0.0002) prevalence of *H. ursi* infection than adult male ticks (50.0%; Table 7). However, there were no significant differences in *H. ursi* infection rates among ticks from different locations or life stages. Additionally, ticks on bears captured in DSY (84.0%) had a significantly higher (*P* = 0.0014) *Babesia* spp. infection rate than those captured in YNP (50.70%; Table 7). However, there was no significant difference in *Babesia* infection rates among ticks of different sexes or life stages.

### The relationships between Formosan black bears’ status and their ticks carrying *H. ursi* and *Babesia* spp.

For each individual bear, the blood parasite infection status, numbers and species of ticks, and proportion of ticks harboring blood parasites are shown in Table 8. Ticks were collected from 13 of 21 (56.52%) bears. Notably, each bear was infested with up to four tick species per time (Table 6). *H. flava* was the predominant tick species (49.16%) infesting wild Formosan black bears in Taiwan, whereas the prevalence of the other tick species was below 20% (Table 4). Regardless of whether individual bears were infected with *H. ursi* and/or *Babesia* spp., the infesting ticks harbored blood parasites (Table 8).

**Table 8:** Numbers of ticks infesting bears, diversity of ticks, and *H. ursi* and *Babesia* spp. detected in wild Formosan black bears and ticks

Bear number	*H. ursi* PCR^*^	*Babesia* spp. PCR	Number of ticks infesting bears (*n* = 240)	Number of ticks positive for *H. ursi* (%) (*n* = 157)	Number of ticks positive for *Babesia* spp. (%) (*n* = 128)
BB01	+	+	34 *H. flava*	28 (82.35)	20 (58.82)
BB02	+	+	19 *H. flava*	15 (78.94)	10 (52.63)
			1 *I. acutitarsus*	1 (100.0)	1 (100.0)
			1 *D. taiwanensis*	1 (100.0)	1 (100.0)
			1 *Haemaphysalis* spp.	1 (100.0)	1 (100.0)
BB03	+	+	12 *H. flava*	10 (83.33)	9 (75.0)
			1 *D. taiwanensis*	1 (100.0)	1 (100.0)
BB04	+	+	18 *H. flava*	18 (100.0)	12 (66.67)
			9 *Haemaphysalis* spp.	8 (88.89)	7 (77.78)
			1 unidentified	1 (100.0)	1 (100.0)
BB05	+	+	13 *H. hystricis*	10 (76.92)	8 (61.53)
			1 *A. javanense*	1 (100.0)	1 (100.0)
BB06	−	+	10 *I. ovatus*	10 (100.0)	6 (60.0)
			2 *D. taiwanensis*	2 (100.0)	2 (100.0)
			1 *H. hystricis*	1 (100.0)	1 (100.0)
BB07	+	−	19 *H. flava*	15 (79.0)	13 (68.42)
			11 *I. ovatus*	5 (45.45)	4 (36.36)
			5 *Haemaphysalis* spp.	3 (60.0)	5 (100.0)
			1 *I. acutitarsus*	1 (100.0)	0 (0)
BB08	−	−	2 *D. taiwanensis*	2 (100.0)	2 (100.0)
			1 *H. hystricis*	1 (100.0)	1 (100.0)
			1 *H. flava*	0 (0)	1 (100.0)
BB09	+	−	20 *A. testudinarium*	6 (30.0)	3 (15.0)
			6 *H. longicornis*	3 (50.0)	0 (0)
			6 *H. hystricis*	2 (33.33)	0 (0)
			4 *H. flava*	0 (0)	4 (100.0)
			2 *Haemaphysalis* spp.	2 (100.0)	0 (0)
BB10	+	+	8 *H. hystricis*	0 (0)	0 (0)
			5 *A. testudinarium*	1 (20.0)	4 (80.0)
			2 *D. taiwanensis*	0 (0)	2 (100.0)
BB11	−	+	7 *H. hystricis*	1 (14.28)	7 (100.0)
			1 *D. taiwanensis*	1 (100.0)	1 (100.0)
BB19	+	−	9 *H. flava*	3 (33.3)	0 (0)
			1 *H. hystricis*	0 (0)	0 (0)
BB20	+	−	3 *H. hystricis*	3 (100.0)	0 (0)
			2 *H. flava*	0 (0)	0 (0)

^*^+, PCR-positive; −, PCR-negative

Ticks collected from *Babesia*-positive bears were more likely (*P* < 0.00001) to harbor *Babesia* spp. than those collected from *Babesia*-negative bears [65.30% (96/147) versus 34.40% (32/93)]. However, there were no significant differences in *H. ursi* detection rates between ticks collected from *H. ursi*-positive and *H. ursi*-negative bears.

## Discussion

To the best of our knowledge, this is the first study to establish a hematological and plasma biochemical database of free-ranging black bears in Taiwan and investigate ectoparasite infestation, blood parasites, and tick-borne pathogen. In this study, there were no significant differences in hematological values among the different sexes or ages. Notably, the median values of PCV, RBC, Hb, MCV, and other erythrocyte-related parameters in the present study and in wild American black bears were lower than those of captive Formosan black bears [13] and other Asian black bears [14], which may be due to a richer diet in captive environments [49, 50]. However, erythrocyte-related values in wild Formosan black bears were lower than those in wild American black bears [25, 50]. Overall, these differences in hematological values could be species-specific or caused by differences in food resources.

Additionally, plasma biochemical parameters were significantly affected by sex. For example, TPP, creatinine, Ca^2+^, Mg^2+^, and K^+^ levels were significantly higher in male bears than in female bears; however, the reason for this remains unknown. TPP is an indicator of dietary protein levels and is correlated with the primary food sources of bears [51]. Additionally, the lower levels of Ca^2+^ in female bears than in male bears may be attributed to long-term pregnancy and lactation [50, 52]. In the present study, pathogen-infected bears had higher levels of inflammatory indicators, including ESR (30 min and 1 h) and globulin than uninfected bears. Moreover, there were significant differences in ESR (1 h), albumin, globulin, A/G, ALP, and Ca^2+^ levels between uninfected and pathogen-infected bears. Furthermore, Wright–Giemsa staining indicated the presence of *Hepatozoon* spp. gamonts in the blood of bears with high ESR and globulin values. Notably, changes in albumin, globulin, and ALP levels have also observed in other *Babesia*-infected hosts, such as camels (*Camelus dromedarius*) [53], dogs (*Canis familiaris*) [54], and cattle [55] as well as *Hepatozoon*-infected dogs [56–58]. Collectively, these hematological and plasma data may serve as a reference for future investigation on host and tick-borne pathogen relationships in wild bears.

Importantly, the differences in capture procedures also seemed to affect the blood profile of the bears. Consistent with previous findings in wild European brown bears and American black bears [28, 59], WBC, segments, CK, and LDH values were elevated in snare-captured bears, which may be attributed to potential stress and muscle injuries. However, diagnosing muscle injuries in bears requires physical examinations and imaging, which can be challenging in the field. Information based on satellite tracking and blood testing results seemingly can only reveal temporary muscle injuries in snare-captured bears. Therefore, we suggest that, when circumstances warrant, traps should be considered as a priority. Otherwise, snare traps should only be applied in remote areas with poor accessibility or emergency conditions, and when traps can be monitored in real time or checked daily.

Currently, 5 species of soft ticks and 44 species of hard ticks have been recorded in Taiwan [60–62]. Among the eight species of hard ticks found in wild Formosan black bears in this study, only *A. javanense* has not been previously recorded in Taiwan. *H. flava* appears to be the predominant tick infesting wild Formosan black bears, and has also been found in several mammals in Taiwan, including wild boars (*Sus scrofa*), deer (*Rusa unicolor*), and dogs [63]. Additionally, *H. flava*, *A. testudinarium*, *H. longicornis*, and *D. taiwanensis* have been recorded in wild Japanese black bears (*U. t. japanicus*) [17, 23]. *A. javanense* ticks are commonly found in reptiles and mammals, especially in pangolins [64]. Phylogenetic analysis and data of tick species in other animals in the same area would be helpful in investigating the distribution and sources of *A. javanense* in the country.

To the best of our knowledge, this is the first study to report *H. ursi* and *Babesia* spp. in wild Formosan black bears and their ticks. Notably, *H. ursi* infection had a lower prevalence in Formosan black bears (61.90%) than in Japanese black bears (76.3% and 100%) [17, 43], Indian sloth bears (*Melursus ursinus*; 70%) [18], and Turkish brown bears (*Ursus arctos*; 100%) [65]. Additionally, *H. ursi* detected in this study had a 99.1–99.7% nucleotide similarity with isolates from Japanese black bears (EU041718). Currently, *H. ursi* is the only *Hepatozoon* spp. detected in the family Ursidae [66]. Although wild Asiatic black bears have high *H. ursi* prevalence, there are no current reports on *Hepatozoon* spp. in other bear species such as American black bears, European brown bears, and polar bears [42, 67, 68], which may be because the suspected vector, *H. flava*, is mainly distributed in Asian countries [17, 69–72].

*Hepatozoon* spp. can be transmitted to hosts via ingestion of sporulated oocysts in ticks or other arthropod vectors [73–75]. In addition to the ingestion of vectors, predators, such as wild canids that hunt grey squirrels harboring the cystozoite stage of *Hepatozoon* spp., have also been suggested as a possible transmission route [76–78]. *Hepatozoon* infection in wild carnivores, such as the red fox (*Vulpes vulpes*) [79], European wild cat (*Felis silvestris silvestris*) [80], and pine martens (*Martes martes*) [81], usually cause little harm to the host. However, *Hepatozoon*-infected wildlife, which play an important role in the circulation of this pathogen, may represent a complementary natural reservoir for domestic animals [82]. For example, immature and mature meronts of *Hepatozoon* were histopathologically identified in the lungs of *Hepatozoon*-infected Japanese black bears [66]. Additionally, the lungs of these bears may harbor the schizogonic developmental stages of *H. ursi* [17].

Infections with *Babesia* spp. have been previously reported in Japanese and American black bears [43, 83–85]. In the present study, *Babesia* spp. infecting Formosan black bears were closely related to other *Babesia* species, such as *B. gibsoni* from dogs, *B. microti* from foxes (*Vulpes* spp.), and *B. odocoilei* from elks (*Cervus elaphus canadensis*). Similarly, *Babesia* DNA from American black bears was closely related to sequences from raccoons or domestic dogs [83]. Collectively, these results indicate that black bears can be infected by several *Babesia* spp. Furthermore, the unidentified *Babesia* spp. found in this study showed a 90.6–95.5% sequence similarity with known *Babesia* spp., highlighting the importance of conducting additional amplification and sequencing of the full-length 18S rRNA gene or other specific genes of *Babesia* to achieve a more detailed genetic characterization.

*Babesia* spp. are commonly transmitted to hosts by competent feeding ticks, followed by transfusion of infected blood products and vertical transmission [86, 87]. *Babesia* infections are typically asymptomatic in wild animals [88, 89]. For example, the clinical impact of babesiosis in bears is limited, and only a single episode of anemia has been reported in a *Babesia*-infected Japanese brown bear (*U. t. japonicus*) heavily infested with ticks [90]. However, the role of black bears as potential wildlife reservoirs of *Babesia* and other vector-borne pathogens warrants further investigation.

Although sex, age, or origin did not significantly affect the prevalence of blood parasites related to sex, age, or location, the prevalence of *H. ursi* infection was significantly higher in bears captured in YNP (13/14; 92.85%) than in those captured in DSY (0/7; 0%). *H. ursi* seemed to dynamically circulate among ticks, bears, and the environment in the YNP, but not in the DSY. Among the tick species that harbored *Hepatozoon* spp., *H. flava* is likely the most epidemiologically relevant. In Japan, mature *Hepatozoon* oocysts have been found in *H. flava* and *H. japonica* collected from dead Japanese black bears [17]. Overall, these findings indicate that *H. flava* plays an important role in *Hepatozoon* transmission to wild black bears. However, the vector competence of *H. flava* for *Hepatozoon* transmission requires further study. In the present study, female ticks had a higher prevalence of *H. ursi* infection than male ticks. Unlike male ticks, female ticks have a larger physiological demand for blood because they require it for engorgement prior to egg laying. This engorgement process provides them with additional opportunities to acquire pathogens from the host.

In Taiwan, *Dermacentor taiwanensis* and *H. flava* show a high prevalence of *Babesia* infections. *Dermacentor taiwanensis* infests several wild animals, including boar (*Sus scrofa taivanus*), bamboo partridge (*Bambusicola thoracica*), murine rodents (*Bandicota*, *Rattus*, *Mus*), tree squirrel (*Callosciurus*), hares (*Lepus*), and mustelid and viverrid carnivores (*Mustela*, *Melogale*, *Paguma*) [91]. In the present study, BLAST analysis revealed that *Babesia* DNA samples from ticks matched several species of *Babesia*. Although the species of *Babesia* was not identified due to the wide host range of ticks, we showed that ticks that infest Formosan black bears may be infected by several species of *Babesia*. Additionally, ticks on bears captured in DSY were more likely to harbor *Babesia* spp. than those captured in YNP. To further clarify whether the location affects the prevalence of *Babesia* infection in vectors, *Babesia* should be detected in ticks from the same environment.

Notably, regardless of whether the individual bears were infected with *H. ursi* and/or *Babesia* spp., collected ticks harbored blood parasites. Additionally, ticks collected from *Babesia*-positive bears were more likely to harbor *Babesia* spp. than ticks collected from *Babesia*-negative individuals. Importantly, the collected ticks harbored blood parasites even in bears that were not infected for several reasons, such as meal contamination (current or previous blood meals) and environmental contamination (on tick or host surfaces) [92]. Bears and ticks inhabit environments with a high prevalence of blood parasites, allowing ticks to carry these blood parasites [92]. Considering that previous research on *Babesia* spp. has rarely mentioned the relationship between host infection status and infesting vectors, this finding provides information on the association between *Babesia* spp.-infected ticks and Formosan black bears. Although only *H. ursi* and *Babesia* spp. were detected in wild Formosan black bears and in infesting ticks in this study, the other investigated blood parasites and pathogens such as *D. ursi*, *Bartonella* spp., *Rickettsia* spp., *B. burgdorferi* s.l., *Ehrlichia chaffeensis*, *Anaplasma phagocytophilum*, and *Toxoplasma gondii* have been reported in Japanese and American black bears [16, 20, 26, 93, 94]. Overall, the different results can be attributed to various factors, including geographical location, host specificity, tick species diversity, or sampling methodology. Comprehensive epidemiological surveys of parasites and other pathogens, particularly of endangered species, are recommended.

*Amblyomma testudinarium*, *I. acutitarsus*, and *Rhipicephalus sanguineus* s.l. ticks are frequently found in humans in Taiwan, followed by *H. hystricis* and *Rhipicephalus haemaphysaloides* [95–97]. These ticks harbor and act as potential vectors for zoonotic and human pathogens. In November 2019, a single human case of “severe fever with thrombocytopenia syndrome” (SFTS) was reported in Taiwan, resulting in death 40 days after the onset of clinical signs [98]. The tick-borne SFTS virus can be transmitted by *H. longicornis* [99], and has also been detected in *A. testudinarium* in Korea [100]. In Taiwan, the SFTS virus has been identified in serum samples collected from sheep, cattle, and dogs, and in *R. microplus* ticks collected from cattle [101]. Additionally, the Oz virus, a novel *Thogotovirus* that causes febrile illness and death in humans, was first isolated from *A. testudinarium* in Japan [102]. Overall, these findings emphasize the potential risk of zoonotic pathogen transmission. In the present study, both *H. longicornis* and *A. testudinarium *infested Formosan black bears. Additionally, both species may potentially infest humans and increase the risk of zoonotic diseases, such as SFTS and febrile illness caused by the Oz virus in Taiwan.

## Conclusions

In this study, we established a database of the hematological and plasma profiles of free-ranging Formosan black bears in Taiwan and investigated the occurrence of ectoparasites, blood parasites, and vector-borne pathogens to elucidate the health status and pathogen dynamics within the bear population in Taiwan. Notably, a regional hematological and plasma biochemical database across various subspecies of Asiatic black bears in 18 countries [103] may serve as an essential resource for wildlife veterinarians and biologists in disease diagnosis and monitoring.

### Supplementary Information

Additional file 1: Table S1. Information regarding the 21 wild Formosan black bears sampled from 2014 to 2021.

## Data Availability

All data generated or analyzed during this study are included in this published article.
