# Supplementary material for: Hematological and plasma profiles and ticks and tick-borne pathogens in wild Formosan black bears (Ursus thibetanus formosanus)
Source: Parasit Vectors. 2024 May 28;17:241. doi: 10.1186/s13071-024-06320-7 (PMC11131195; doi:10.1186/s13071-024-06320-7)
Supplement: Supplementary file 1 — Additional file 1: Table S1. Information regarding the 21 wild Formosan black bears sampled from 2014 to 2021. [file 13071_2024_6320_MOESM1_ESM.docx]

**Table S1.** Information regarding the 21 wild Formosan black bears sampled from 2014 to 2021.

| **Bear No.** | **Sex** | **Age** | **Area** | **Trap** | **Sampling date**  **(DD/MM/YYYY)** | **No. of collected ectoparasites** |
| --- | --- | --- | --- | --- | --- | --- |
|  |  |  |  |  |  |  |
| BB01 | Female | Adult | YNP | Snare | 10/11/2014 | 34 |
| BB02 | Female | Adult | YNP | Snare | 11/11/2014 | 22 |
| BB03 | Male | Adult | YNP | Snare | 19/11/2014 | 13 |
| BB04 | Female | Adult | YNP | Snare | 14/12/2014 | 28 |
| BB05 | Male | Adult | YNP | Culvert | 30/06/2015 | 14 |
| BB06 | Female | Adult | DSY | Culvert | 26/11/2015 | 13 |
| BB07 | Female | Adult | YNP | Snare | 20/12/2015 | 36 |
| BB08 | Male | Subadult | DSY | Culvert | 21/12/2015 | 4 |
| BB09 | Female | Adult | YNP | Culvert | 30/04/2016 | 38 |
| BB10 | Female | Adult | YNP | Culvert | 16/05/2016 | 15 |
| BB11 | Male | Adult | DSY | Culvert | 19/05/2016 | 8 |
| BB12 | Female | Adult | DSY | Culvert | 16/10/2016 | * |
| BB13 | Male | Adult | DSY | Culvert | 26/11/2018 | * |
| BB14 | Female | Adult | DSY | Culvert | 20/12/2018 | * |
| BB15 | Male | Adult | DSY | Culvert | 28/03/2019 | * |
| BB16 | Male | Subadult | YNP | Culvert | 13/11/2020 | * |
| BB17 | Male | Adult | YNP | Culvert | 20/03/2021 | * |
| BB18 | Male | Adult | YNP | Culvert | 26/04/2021 | * |
| BB19 | Male | Adult | YNP | Culvert | 03/05/2021 | 10 |
| BB20 | Male | Adult | YNP | Culvert | 02/06/2021 | 5 |
| BB21 | Male | Adult | YNP | Culvert | 12/07/2021 | * |

*No ectoparasite was observed by the naked eye.
